# Relative risk of renal disease among people living with HIV: a systematic review and meta-analysis

**DOI:** 10.1186/1471-2458-12-234

**Published:** 2012-03-23

**Authors:** Fakhrul M Islam, Jianyun Wu, James Jansson, David P Wilson

**Affiliations:** 1 The Kirby Institute, University of New South Wales, Sydney, NSW 2052, Australia; 2 Corner of West and Boundary Streets, Darlinghurst, Sydney, NSW, Australia

**Keywords:** HIV, Renal disease, Review, Meta-analysis, Relative risk

## Abstract

**Background:**

Antiretroviral therapy (ART) has substantially decreased mortality and HIV-related morbidity. However, other morbidities appear to be more common among PLHIV than in the general population. This study aimed to estimate the relative risk of renal disease among people living with HIV (PLHIV) compared to the HIV-uninfected population.

**Methods:**

We conducted a systematic review and meta-analysis of relative risks of renal disease among populations of PLHIV reported in studies from the peer-reviewed literature. We searched Medline for relevant journal articles published before September 2010, yielding papers published during or after 2002. We also searched conference proceedings of the International AIDS Society (IAS) and Conference on Retroviruses and Opportunistic Infections (CROI) prior to and including 2010. Eligible studies were observational studies reporting renal disease defined as acute or chronic reduced renal function with glomerular filtration rate less than or equal to 60 ml/min/1.73 m^2 ^among HIV-positive adults. Pooled relative risks were calculated for various groupings, including class of ART drugs administered.

**Results:**

The overall relative risk of renal disease was 3.87 (95% CI: 2.85-6.85) among HIV-infected people compared to HIV-uninfected people. The relative risk of renal disease among people with late-stage HIV infection (AIDS) was 3.32 (1.86-5.93) compared to other PLHIV. The relative risk of renal disease among PLHIV who were receiving antiretroviral therapy (ART) was 0.54 (0.29-0.99) compared to treatment-naïve PLHIV; the relative risk of renal disease among PLHIV who were treated with tenofovir was 1.56 (0.83-2.93) compared to PLHIV who were treated with non-tenofovir therapy. The risk of renal disease was also found to significantly increase with age.

**Conclusion:**

PLHIV are at increased risk of renal disease, with greater risk at later stages of infection and at older ages. ART prolongs survival and decreases the risk of renal disease. However, less reduction in renal disease risk occurs for Tenofovir-containing ART than for other regimens.

## Background

Highly active antiretroviral treatment (ART) has reduced mortality and decreased HIV-related morbidities, such that people living with HIV (PLHIV) are living longer, healthier lives [1]. However, PLHIV face other health challenges. Comorbid conditions are becoming more important in the health care of PLHIV [1]. Numerous serious morbidities are more likely to occur among PLHIV than among the general population. It is important to understand the incidence rate of these comorbidities among PLHIV, along with their risk factors, in order to guide clinical care and planning of health systems.

Chronic kidney disease (CKD) is increasing worldwide and the ensuing end stage renal disease necessitates transplantation or dialysis, both of which are highly expensive [1]. There is no consensus on the risk of renal dysfunction associated with HIV infection and the use of ART, however, it is believed to be an important condition disproportionately affecting PLHIV [2-4]. Numerous studies have investigated the rates of renal disease and its risk factors for PLHIV. In this study we conduct a systematic review and meta-analysis to assess the relative risk of renal disease among PLHIV.

## Methods

### Search strategy

We conducted a comprehensive literature search of the published literature through Medline with the following key words: 'HIV or human immunodeficiency virus or AIDS or acquired immunodeficiency syndrome' AND 'renal failure or kidney failure or renal impairment or kidney impairment or renal insufficiency or kidney insufficiency or renal disease or kidney disease or acute kidney injury or glomerulonephritis or GN or nephropathy or proteinuria or nephritic sediment or hematuria or erythrocyturia or leukocyturia or glucosuria or tubulotoxic damage or Fanconi's syndrome or HIV-AN or HIVAN or dialysis or hemodialysis or peritoneal dialysis or ESKD or ESRF or ESKF' AND 'relative risk or risk ratio or RR or odds ratio or OR or hazard ratio or HR or incidence'. We also searched abstracts from CROI and International AIDS Society (IAS) conferences during years 2000-2010, with the same search terms.

### Outcome measures

The primary outcome for our analysis was chronic kidney disease (CKD) defined as estimated glomerular filtration rate or eGFR < 60 mL/min/1.73 m^2 ^for greater than or equal to 3 months irrespective of kidney damage [5]. An eGFR less than 60 mL/min/1.73 m^2 ^for less than 3 months refers to acute renal failure (ARF). The rationale for the inclusion of individuals with eGFR less than 60 mL/min/1.73 m^2 ^without any other evidence of kidney damage is that at least 50% of normal kidney function decreases and the prevalence of complications of CKD begins to increase below this level [6]. In addition, abnormal proteinuria is more likely to occur at this level, which is an indication of kidney disease [7]. It is noted here that there are five stages of CKD, which are defined as stage 1: eGFR > 90 (normal); stage 2: eGFR: 60-89 (mild), stage 3: 30-59 (moderate); stage 4: 15-29 (severe); and stage 5: eGFR < 15 (kidney failure) [5]. There is a complex spectrum of renal diseases, such as HIV-associated nephropathy (HIVAN), end-stage renal disease (ESRD), all-cause nephropathy (ACN), acute renal failure (ARF), chronic kidney disease (CKD), chronic renal failure (CRF), tubular dysfunction, and renal impairment (RI), which can be classified within CKD stages 3, 4 and 5 based on the outcome measure in each individual study. Our review includes stage 3 (moderate kidney damage) to stage 5 (kidney failure) CKD. We included any study that reported an incidence rate ratio (IRR), relative risk (RR), odds ratio (OR) or hazard ratio (HR) of the renal events among PLHIV.

### Selection of studies

**Figure 1:**
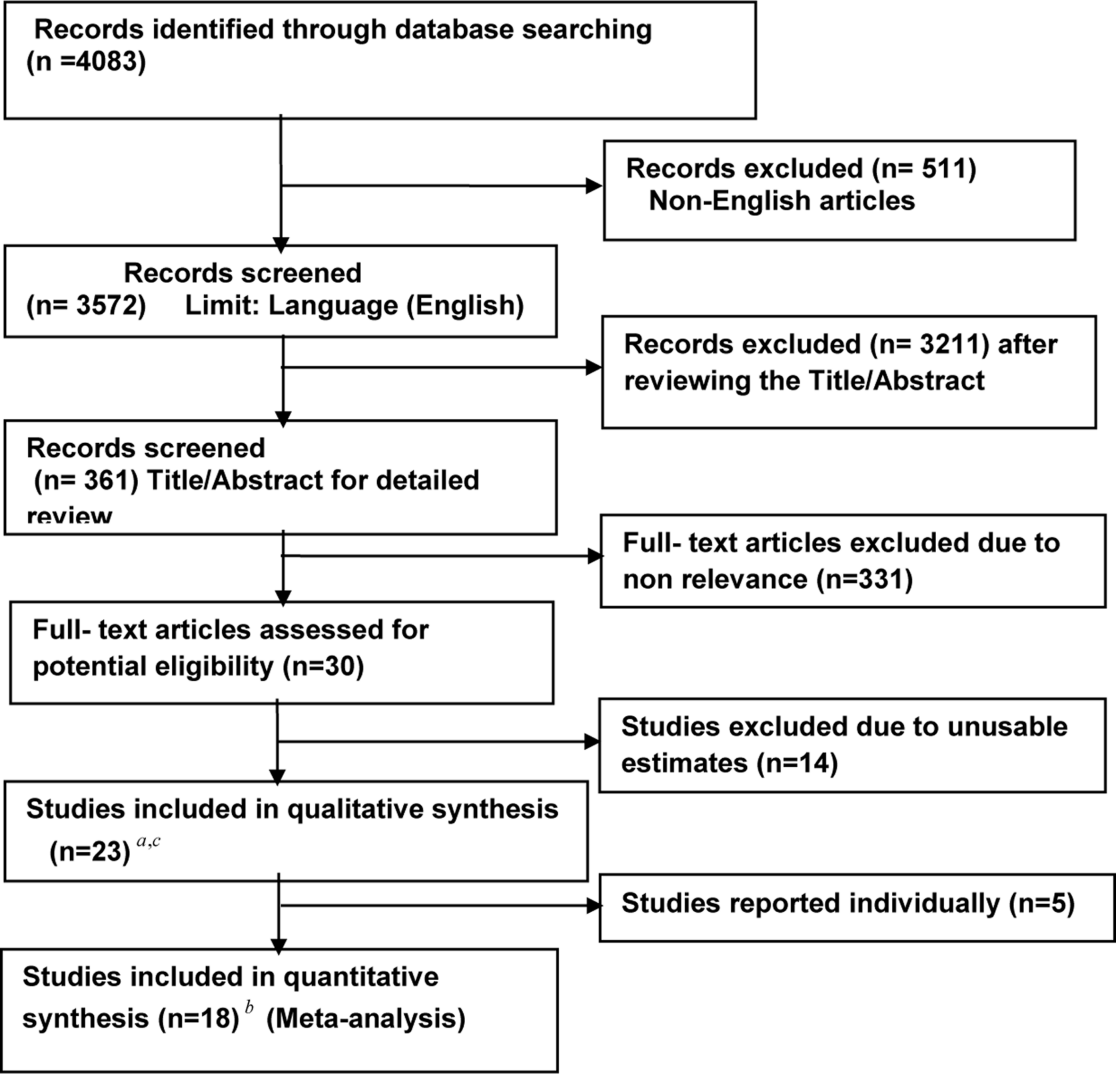
**Information flow diagram of the systematic review**. **a **Qualitative synthesis: results of primary studies are summarized but not statistically combined; **b **Quantitative synthesis: Statistical methods applied to combine the results of two or more studies **c **includes conference proceedings (7).

Two reviewers independently assessed each potential relevant article for eligibility (FI, JW). Disagreements were resolved with other authors until a consensus was reached. We screened the titles and the abstracts of the 4083 articles, identified by the search phrases, for appropriateness before retrieval of the full-text. Studies that reported HIV and/or AIDS and renal disease, and provided estimates of risk factors were included in the analysis. Studies that did not report the risk estimates were excluded from further review. Non-English and review articles were also excluded from the analysis. The selection process for studies included in the formal analysis [2-4,7-26], is presented in Figure 1.

### Data extraction

Two reviewers independently extracted data using a standardized form. The information recorded from each study were author, study design, study types, study period, publication year, follow-up period, sample size, renal disease, eGFR or other measure, comparator groups, reported outcome, risk estimates, age, sex, race/ethnicity and geographic location. The details of the data extraction are listed in Table 1.

**Table 1:** Details of study populations

Study name	Study Type (Cohort name)		Study period	Age (Mean) of participants	Sex (Male (%))	Primary outcome(Disease)	Location	Study size(N)		Race/ethnicity (%)	Average follow up(years)	Total Events	Method for eGFR (< 60 ml/min/1.73m^2^)
Atta, 2006 [8]	Cohort		1995-2004	42.8	58	HIVAN	MD, USA	263		96(B)	8.0	36	MDRD
Buskin, 2009 [9]	Cohort		1996-2003	36.5		HIVAN	USA	59705		6.2(W)	3.5	5042	MDRD
										11.5(B)			
										7.3(H/L)			
										6.5(A/PI)			
										9.7(NA/AN)			
										4.7(O)			
Campbell, 2009 [10]	cross-sectional		1998-2005		--	CKD	BT, UK	3439		NR	--	81	MDRD
Crane, 2007 [4]	Cohort(UWHIV)		2001-2006	41	83	Kidney dysfunction	WA, USA	445		61(W)	--	51	CG
										23(AA)			
										16(O)			
Crum-Cianflone, 2010 [11]	cross-sectional		2004-2005	41	92	Kidney dysfunction	CA, MD, USA	717		48.7 (C)	--	22	MDRD
										37.9(AA)			
										13.4 (O)			
Deti, 2010 [7]	Cohort(ANRS CO3)		2004-2008	43	75	CRF	France	2613		NR	3.4		MDRD
Franceschini, 2006 [12]	Cohort		2000-2002	40	69	ARF	NC, USA	705		66(AA)	3.0		serum creatinine
Franey, 2009 [13]	Cohort		2004-2007	36	31.2	RI	South Africa	2189		NR	--	287	MDRD
Ganesan, 2010 [14]	Cohort (P)		1986-2008	29	92	CKD	USA	4044		45(AA)	6.5	90	MDRD
										44(EA)			
Heffelfinger, 2006 [15]	Cohort		2000-2003	--	--	RI	USA	11362		NR		724	serum creatinine
Horberg, 2010 [3]	Cohort(KP)		2002-2005	43	86.1	Tubular dysfunction	CA, ML, VA, USA	1647		24.5(B)	2.0		MDRD
Ibrahim, 2010 [2]	Cohort(KCH)		1999-2008	37	62	ARF	SL, UK	2556		55.4(B)	--	184	MDRD
										44.6(W/O)			
Jacobson, 2007 [16]	Cohort(MACS)					Proteinuria	USA	1203		(B)		120	
Jones, 2004 [17]	Cohort(CWS)					Renal dysfunction	UK	4183		NR		1175	serum creatinine > 120
Krawczyk, 2004 [18]	Cohort(HOPS)		1992-2002	43	82.5	CRF	GA, USA	6361		43.8(W/NH)		108	--
										5.0(H)			
										48.8(B)			
										2.5(O)			
Longenecker, 2009 [19]	Cohort(P)		2004-2007	41	68	CKD	CA, USA	554		42(AA)	5.0		CKD-EPI
										48(W)			
										10(O)			
Lucas, 2007 [20]	Cohort(ALIVE/JHHC)		1988-2005	37	68	ESRD/RRT	MD, USA	6255		(AA)	5.7	221	MDRD
Mocroft, 2010 [21]	Cohort(EuroSIDA)		2004-2008	43	75.1	CKD	Europe, Israel, Argentina			85.5(W)	3.7	225	CG
Reisler, 2005 [22]	Cohort(MACS)		2003-2004	--	--	CKD	USA	1470		NR		53	MDRD
													
Roe, 2008 [23]	Cohort (R)		1998-2005	35.9	64	ARF	SEL, UK	2274		64(B)	8.0	130	MDRD
										36(NB)			
Szczech, 2002 [24]	Cohort(WIHS)			40	--			2059		71.4(B)		671.0	double serum creatinine
										16.7(W)			
										11.9(H)			
										0(O)			
Vanig, 2008 [25]	Cohort (R)		2006-2007	--	94	CKD	USA	375		NR			MDRD
													
Wei, 2003 [26]	Cohort(P)			37.6	93.2	HIVAN	NY, USA	44		(AA)		44	

	Study group1					Study group2							

	Study description	Sample size (n1)	Event1	Incidence(per 1000py)		Study description			Sample size (n2)	Event2	Incidence(per 1000py)	HR/RR/OR	95% CI

Study name													
Atta, 2006 [8]	ART	--	26	--		Treatment naïve			--	10	--	0.3	0.09, 0.98
Buskin, 2009 [9]	Indinavir					Treatment naïve						1.15	1.02, 1.29
	Ritonavir					Treatment naïve						0.87	0.77, 0.98
	HAART					Treatment naïve						0.3	0.27, 0.34
Campbell, 2009 [10]	Age > = 50 years(Indinavir)		--	--		Age < 50 years				--	--	4.92	1.31, 18.4
	Indinavir/per year											1.29	1.00, 1.65
	Age > = 50 years(Tenofovir)					Age < 50 years						5.42	1.71, 16.8
Crane, 2007 [4]	Age (30-40) years					Age < 30 years						0.9	0.2, 3.2
	Age (40-50) years					Age < 30 years						2.1	0.6, 7.6
	Age > 50 years					Age < 30 years						4.4	1.1, 17.2
	(NRTI)Didanosine					(NRTI)lamivudine/emtricitabine						3.1	1.40, 6.80
	PI(Amprenavir/fos-amprenavir)					NNRTI (efavirenz)						3.6	1.00, 12.5
Crum-Cianflone, 2010 [11]	Age/10 year increase											1.99	1.22, 3.24
	Tenofovir/per year											1.54	1.10, 2.15
Deti, 2010 [7]	Age/10 year increase											2.2	1.8, 2.6
	CD4 < 200 cells/mm3					CD4 > 500 cells/mm3						4.04	2.3, 7.1
	Tenofovir/per year											1.4	1.1, 1.8
Franceschini, 2006 [12]	CD4 < 200 cells/mm3					CD4 > = 200 cells/mm3						4.7	2.5, 8.8
Franey, 2009 [13]	CD4 < 100 cells/mm3					CD4 > 100 cells/mm3						1.4	1.07, 1.82
	Age > 40 years					Age < 40 years						4.65	3.54, 6.10
Ganesan, 2010 [14]	Age > = 35 years					Age < 35 years						2.6	1.7, 4.0
	CD4 < = 200 cells/mm3					CD4 > = 500 cells/mm3						6.8	3.0, 15.5
	CD4(201-349) cells/mm3					CD4 > = 500 cells/mm4						4.3	2.3, 8.1
	CD4(350-499) cells/mm3					CD4 > = 500 cells/mm5						2.4	1.3, 4.6
Heffelfinger, 2006 [15]	Tenofovir					Treatment naïve						1.5	1.1, 1.9
Horberg, 2010 [3]	Tenofovir	964				Non-Tenofovir			683			5.23	2.08, 13.1
Ibrahim, 2010 [2]	Age/10 year increase											1.04	0.87, 1.24
	Indinavir					Treatment naïve						1.81	0.53, 6.76
	Tenofovir					Treatment naïve						1.06	0.46, 2.43
	Atazanavir					Treatment naïve						1.05	0.20, 5.49
	CART					Treatment naïve						1.11	0.54, 2.30
	CD4(201-350) cells/mm3					CD4 > 350 cells/mm3						1.56	0.97, 2.48
	CD4(101-200) cells/mm3					CD4 > 350 cells/mm3						2.08	1.11, 3.91
	CD4(51-100) cells/mm3					CD4 > 350 cells/mm3						6.38	3.18, 12.78
	CD4(0-50) cells/mm3					CD4 > 350 cells/mm3						10.29	5.11, 20.98
Jacobson, 2007 [16]	HIV	94				Non-HIV			26			5.1	2.9, 8.9
Jones, 2004 [17]	Tenofovir	1058	84			Treatment naïve						0.22	0.07, 0.69
Krawczyk, 2004 [18]	CD4 < 200 cells/mm3					200-349 cells/mm3						4.3	2.1, 8.7
	HAART					Treatment naïve						0.5	0.3, 1.0
Longenecker, 2009 [19]	HIV	337	26			Non-HIV			230	2		6.5	1.5, 28.7
	Age/10 year increase											1.34	1.02, 1.77
Lucas, 2007 [20]	AIDS	1902	125	12.7		No AIDS			2607	51	4.7	2.7	1.9, 4.0
	Age(30-49.9)					Age < 30 years						1.5	0.7, 3.3
	Age > = 50 years					Age < 30 years						2	0.8, 4.7
	AIDS	1902				Non-HIV			1751			5.1	3.5, 7.6
	HIV	2607				Non-HIV			1751			2.3	1.5, 3.5
Mocroft, 2010 [21]	AIDS					No AIDS						2.22	1.14, 4.32
	Age/10 year increase											1.54	1.31, 1.80
	Tenofovir/per year											1.16	1.06, 1.25
	Indinavir/per year											1.12	1.06, 1.18
	Atazanavir/per year											1.21	1.09, 1.34
	Lopinavir/r per year											1.08	1.01, 1.16
Reisler, 2005 [22]	HIV		32			Non-HIV				21		2.5	1.4, 4.5
	Tenofovir					Non-Tenofovir						2	0.8, 4.9
Roe, 2008 [23]	CD4 < 100 cells/mm3					CD4 > 200 cells/mm3						6.75	2.5, 18.3
	CD4(100-199) cells/mm3					CD4 > 200 cells/mm3						3.02	0.99, 9.13
	AIDS					No AIDS						6.7	3.4, 13.3
Szczech, 2002 [24]	CD4 < = 200 cells/mm3					CD4 > 200 cells/mm3						3.57	1.72, 7.14
Vanig, 2008 [25]	Tenofovir	253				Non-Tenofovir			122			1.83	1.11, 3.02
	PI					Non-PI						1.27	0.84, 1.91
Wei, 2003 [26]	ART					Treatment naïve						1.51	0.37, 6.20
	CD4 < = 100 s cell/mm3					CD4 > = 100 cells/mm3						2.73	0.82, 9.14

A, Asian; AA, African-American; B, Black; EA, European American; H, Hispanic; NH, Non-Hispanic; L, Latino; NB, Non-b lack; PI, Pacific Islander; NA, Native American; AN, Alaska Native; W, White; O, Others; MD, Maryland; NC, North Carolina; WA, Washington; GA, Georgia; SL South London; SEL, Southeast London; BT, Brighton; NR, Not Reported; NA, Not Applicable; Sr, Serum; R, Retrospective; P, Prospective; WIHS, Women's Interagency HIV Study; UWHIV, University of Washington HIV; ALIVE, AIDS Link to the Intravenous Experience; JHHC, Johns Hopkins HIV Cohort; KP, Kaiser Permanente; MACS, Multicentre AIDS Cohort Study; CWS, Chelsea and Westminster; HOPS, HIV Outpatient Study; KCH, Kings College Hospital; ANRS CO3, National agency for AIDS research Aquitaine Cohort; MDRD, Modification of Diet in Renal Disease-an equation to estimate GFR that incorporates serum creatinine, age, sex and race; CG, Cockcroft-Gault equation to estimate GFR that incorporates serum creatinine, age, sex and weight; CKD-EPI, Chronic Kidney Disease Epidemiology Collaboration-an equation that take into account serum creatinine, age, sex and race.

### Methods for assessing renal function outcomes

The identified studies measured the change in renal function by various methods: fourteen studies reported measuring eGFR using an MDRD method, two studies using the CG formula, one used the CKD-EPI formula and six studies reported renal dysfunction measured by serum creatinine (see Table 1). Regardless of method, studies reporting eGFR less than 60 mL/min/1.73 m^2 ^(at least moderate CKD) were included for further analysis.

### Quality assessment of included studies

Two reviewers independently rated the quality of each study using the Downs and Black checklist [27] The checklist comprises of 27 criteria, including (10) types of reporting, (3) checks of external validity for the generalisability of study population, (7) assessments of bias, (6) exploration of confounding and (1) power. Using this checklist, we calculated an average quality index score of our 23 non-randomized studies to be 14.9, with a range of 11.5 to 19.

### Statistical analysis

We performed a series of meta-analyses based on similar comparator groups among the studies. The risk estimates extracted from the publications were either from logistic regression or proportional hazards models with reported confidence intervals. The extracted estimates were already adjusted for common risk factors in each individual study, such as age, sex, race, smoking, diabetes and hypertension. The rational to pool relative risks from these two types of the models was based on the investigation of D'Agostino et al. [28]. D'Agostino et al. demonstrated the asymptotic equivalence of estimating relative risks from logistic regression and proportional hazards models. Along with the meta-analysis publication by Lollgen et al. [29] that adopted this approach, we concluded that it is reasonable to pool relative risks from these two different models. We calculated the pooled estimates of risks for groups in which there were at least two individual studies. We performed meta-analyses to estimate the pooled relative risk of renal disease among PLHIV compared with HIV-uninfected people; PLHIV on ART (including different classes or specific antiretroviral drugs and duration of ART) compared with PLHIV who are treatment-naïve; people with late-stage HIV (AIDS) compared with other PLHIV; and the effect of age on renal disease. We applied general variance-based methods that used confidence intervals around the effect measures, where all effect types were ratio measures. Outcomes were pooled using the DerSimonian-Laired (DSL) random effects model which accounts for the heterogeneity of the estimates [30]. We quantified the degree of heterogeneity by the I-squared statistic, which can be interpreted as the percentage of total variation across the studies; a value of zero indicates no observed heterogeneity [31]. Secondary analyses were conducted using meta-regression and subgroup analysis.

Two-tailed p-values were considered (p < 0.05) for all statistical tests except for the meta-regression where we considered p < 0.10 to detect potential heterogeneity among covariates. Publication bias was assessed using Egger's method [32]. The analyses of this review were conducted in STATA (version 10; STATA Corporation, College Station, Texas, USA). The methodology and reporting of this review conforms to the Preferred Reporting Items for Systematic Reviews and Meta-Analyses (PRISMA) statement [33,34].

## Results

### Study selection

The search strategy initially resulted in 4083 articles from which we identified 361 for detailed review. After reviewing the titles and abstracts we excluded 331 studies that were not relevant to renal disease among PLHIV. Of 30 articles selected for the potential eligibility, 14 were excluded as they did not relate to our study question. We also searched conference proceedings of Conference on Retroviruses and Opportunistic Infections (CROI) and International AIDS Society prior to and including 2010. Out of the initial 451 results from the conferences, 7 abstracts were selected [7,14-17,22,25]. A total of 23 studies were included for further analysis, two of which were cross-sectional studies, one was a case-control study and twenty were cohort studies. The studies varied greatly with respect to the various comparator groups. Details of the search strategies are listed in Figure 1 and the characteristics of the included studies are presented in Table 1.

### Relative risk of renal disease for PLHIV versus HIV-uninfected people

**Figure 2:**
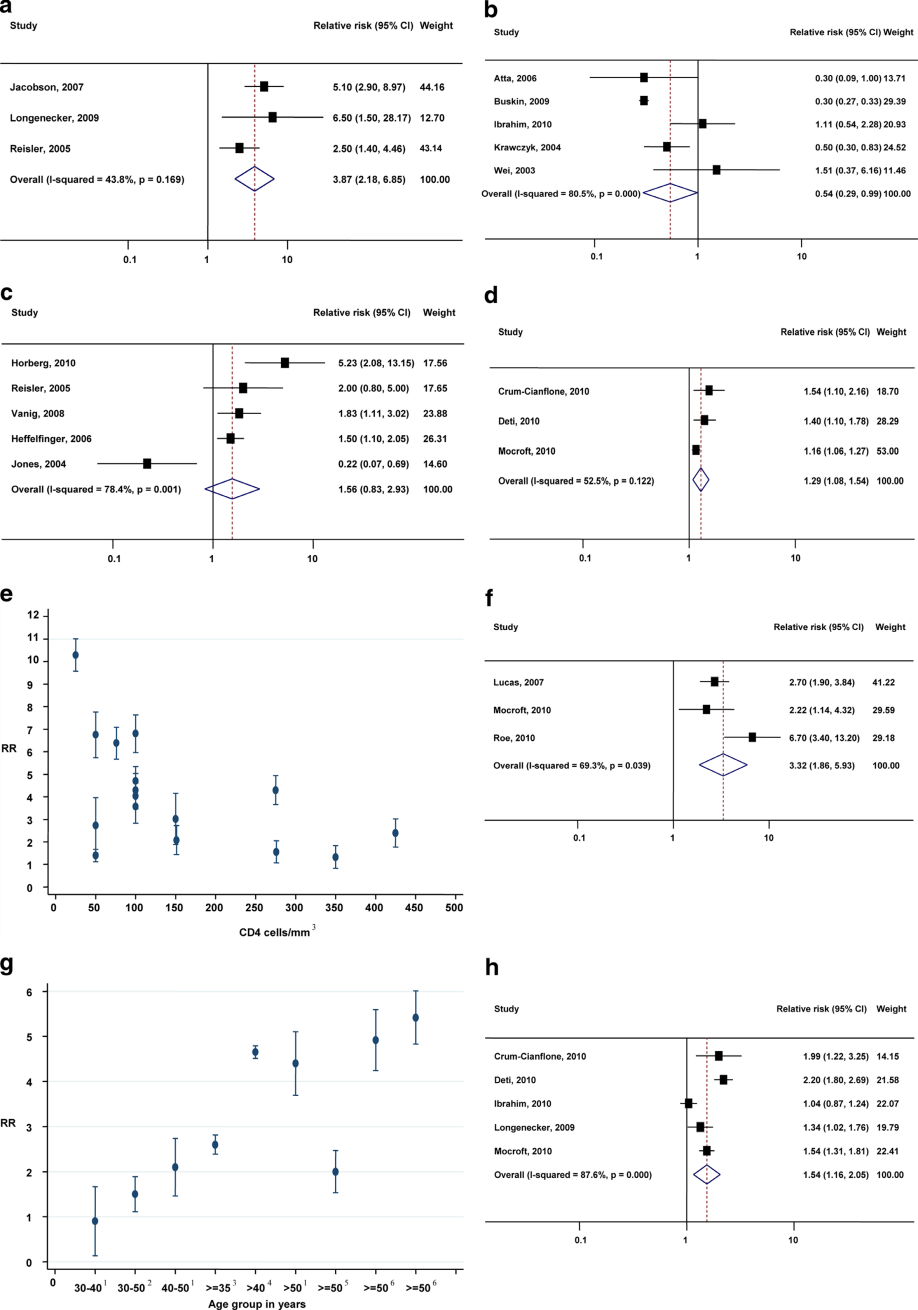
**Forest plot of studies and pooled estimate of relative risk of renal disease**. In **(a)** HIV-infected versus HIV-uninfected people; **(b)** HIV-infected people exposed to antiretroviral treatment versus treatment-naïve PLHIV; **(c)** HIV-infected people exposed to tenofovir-based ART versus non-tenofovir-based ART; **(d)** HIV-infected people per year of exposure to TDF-based ART. **(e)**Relative risk of renal disease in PLHIV according to CD4 count. Mid-values of the range of reported CD4 categories are plotted; each point/bar presents data from a single study, illustrating the RR/95% CI of CKD < 60 for the low CD4 group (CD4 value shown on the abscissa) compared to the high CD4 group (presented in Table 3). **(f)**Forest plot of studies and pooled estimate of relative risk of renal disease among people in AIDS stage versus non-AIDS HIV infection. **(g)**Relative risk of renal disease in HIV-infected people in various age groups (presented in Table 2). ^1^Crane, 2007; compared with < 30, ^2^Lucas, 2007; compared with < 30, ^3^Ganesan, 2010; compared with < 35 ^4^Franey, 2009; compared with < 30; ^5^Lucas, 2007; compared with < 50; ^6^Campbell, 2009; compared with < 50. **(h)**Forest plot of studies and pooled estimate of relative risk of renal disease in HIV-infected people by 10-year increment in age.

Three identified studies reported the risk of kidney disease among PLHIV [16,19,22]. Jacobson et al. reported the relative risk of kidney disease among 542 HIV-infected men with abnormal proteinuria, having CKD stages 3-5, to be 5.1 (95%CI: 2.9-8.9), compared to 661 HIV-seronegative men [16]. This USA-based study recruited adult men and adjusted estimates by age, race, hypertension and diabetes [16]. Another USA-based study, conducted by Longenecker et al [19], compared 337 HIV-infected people (294 ART-experienced) with 230 control subjects. The estimated relative risk of CKD stage 3 among PLHIV was 6.5 (1.5-28.7). The study populations consisted of both male and females aged 41 years or older. The relative risk was adjusted for demographic risk factors such as age, sex and race. Reisler et al. reported the adjusted relative risk of CKD stage 4 among 1470 HIV-infected men compared to HIV-uninfected men to be 2.5 (1.4-4.5) [22]. The pooled relative risk of kidney disease among PLHIV from our meta-analysis was found to be 3.87 (2.18-6.85) compared to HIV-uninfected people (Figure 2a). There was no statistically significant evidence of heterogeneity between the studies (I-squared 43.8%, p = 0.169).

### Effect of antiretroviral treatment

We identified five relevant studies estimating the relative risk of renal failure associated with antiretroviral therapy (ART), compared to treatment-naïve HIV-infected people. Atta et al. reported that the hazard ratio of CKD stage 4 associated with exposure to ART, defined as the initiation of at least one antiretroviral agent, was 0.30 (0.09-0.98). This analysis adjusted for injecting drug users, hepatitis C, GFR and other treatments such as corticosteroids; excluding age, all other characteristics were similar in the two groups [8]. Buskin et al. estimated the adjusted HR of CKD stage 1 to 5 to be 0.30 (0.27-0.34), adjusting for all known risk factors [9]. Of note, 1.1% and 2.9% of patients in this study were CKD stage 1 and 2 respectively and 8.2% of patients were CKD unknown. Ibrahim et al. calculated an adjusted IRR of CKD stage 3 to be 1.11 (0.54-2.30) among HIV-infected people who were exposed to ART not containing indinavir, tenofovir or atazanavir compared with untreated PLHIV [2]. Krawczyk et al. estimated the relative risk of CKD (undefined) to be 0.50 (0.30-1.0) where age, sex and ethnicity were well-matched [18]. Wei et al. estimated the risk ratio of CKD stage 5 among people exposed to ART versus treatment-naïve PLHIV to be 1.51 (0.37-6.2) [26]. We estimated the pooled relative risk across all studies to be 0.54 (0.29-0.99) for renal disease among PLHIV who received ART compared to treatment-naïve PLHIV (Figure 2b). The heterogeneity across these studies was measured to be I-squared = 80.5% (p < 0.001).

### Relative risk of renal disease for tenofovir-based treatment

Some studies have singled out ART containing the antiretroviral drug, tenofovir (TDF), as being an important factor for the incidence of CKD. We collated data from available studies and compared the relative risk of CKD for HIV-infected people treated with TDF-based ART with HIV-infected people receiving non-TDF-based ART. Four cohort studies and one case-control study were relevant for inclusion in this analysis. Horberg et al. reported that the relative risk of CKD stage 3 for people on TDF-based ART was 5.23 (2.08-13.1) compared to people on ART not containing TDF [3]. Reisler et al. reported that the relative risk of CKD stage 3 to 5 using TDF was 2.00 (0.8-4.9) compared to non-TDF-based drugs [22]. Vanig et al. found a relative risk of CKD stage 3 for people receiving TDF-based ART to be 1.83 (1.11-3.02) and Heffelfinger et al. estimated an adjusted odds ratio of CKD stage 3 attributable to TDF of 1.5 (1.1-1.9) [15]. Finally, a case-control study reported by Jones et al. estimated the rate ratio between TDF-containing and non-TDF-based ART to be 0.22 (0.07-0.69) [17]. Pooling the five estimates, we calculated that the overall relative risk of CKD was 1.56 (0.83-2.93) for people on TDF-based ART compared to non-TDF-based ART (Figure 2c). The heterogeneity across these studies was estimated as I-squared = 78.4%, p = 0.001.

### The effect of treatment duration

We investigated whether the duration of exposure to ART influences the risk of CKD. We were able to combine the estimates of three relevant studies [7,11,21]. The combined relative risk of CKD among PLHIV with exposure to TDF-based ART was found to be 1.29 (1.08-1.54) per year of treatment (Figure 2d). There was no strong evidence of heterogeneity between the studies (I-squared = 52.5%, p = 0.122).

### Relative risk of CKD with CD4 ± T-cell-count

Nine studies identified in our review reported that CD4 cell count was associated with the risk of CKD among PLHIV [2,7,12-14,18,23,24,26] (see Table 3). We were unable to combine the estimates in a meta-analysis due to differences in comparator groups (see Table 3 for details of CD4 count categories in all studies). Deti et al. estimated the relative risk of CKD stage 3 among HIV-infected people with CD4 count less than 200 cells/mm^3 ^and between 200-500 cells/mm^3 ^to be 4.04 (2.3-7.1) and 1.3 (0.81-2.2), respectively, compared with those with CD4 count greater than 500 cells/mm^3 ^[7]. Franceschini et al. reported the relative risk of CKD stage 3 for HIV-infected people with CD4 count less than 200 cells/mm^3 ^to be 4.70 (2.5-8.8) compared with those with CD4 count greater than or equal to 200 cells/mm^3^[12]. Franey estimated this risk to be 1.4 (1.1-1.8) with CD4 count less than 100 cells/mm^3^compared to > 100 cells/mm^3 ^[13]. A cohort study reported by Ganesan et al. compared the risk of CKD stage 3 for different CD4 count categories [14]. They found that the relative risks of CKD for HIV-infected people with CD4 count less than or equal to 200 cells/mm^3^, between 201-349 cells/mm^3 ^and between 350-499 cells/mm^3 ^were 6.8 (3.0-15.5), 4.3 (2.3-8.1) and 2.4 (1.3-4.6) compared with people with a CD4 count greater than or equal to 500 cells/mm^3^, respectively. Overall, we found a negative correlation between CD4 count and renal disease (Figure 2e) with lower CD4 counts associated with greater risk of renal disease (correlation, r = -0.52). Other cohort studies support this conclusion, after adjusting for other major confounders [2,18,23,24,26].

**Table 3:** Details of CD4 count categories

Study name	Study type	Period	Diseases	Location	Study size	Follow up	CD4 (group 1) cells/mm3	CD4(group 2) cells/mm3	RR	95%
Deti, 2010	Cohort	2004-2008	CRF	France	2613	3.4	< 200	> 500	4.04	2.3, 7.1
Deti, 2010	Cohort	2004-2008	CRF	France	2613	3.4	200-500	> 500	1.33	0.81, 2.19
Franceschini, 2006	Cohort	2000-2002	ARF	USA	705	3.0	< 200	> 200	4.70	2.5, 8.8
Ganesan, 2010	Cohort	1986-2008	CKD	USA	4044	6.5	< = 200	> = 500	6.8	3.0, 15.5
Ganesan, 2010	Cohort	1986-2008	CKD	USA	4044	6.5	201-349	> = 500	4.3	2.3, 8.1
Ganesan, 2010	Cohort	1986-2008	CKD	USA	4044	6.5	350-499	> = 500	2.4	1.3, 4.6
Franey, 2009	Cohort	2004-2007	RI	South Africa	2189	--	< 100	> 100	1.4	1.07, 1.82
Ibrahim, 2010	Cohort	1999-2008	ARF	UK	2556	--	201-350	> 350	1.56	0.97, 2.48
Ibrahim, 2010	Cohort	1999-2008	ARF	UK	2556	--	101-200	> 350	2.08	1.11, 3.91
Ibrahim, 2010	Cohort	1999-2008	ARF	UK	2556	--	51-100	> 350	6.38	3.18, 12.78
Ibrahim, 2010	Cohort	1999-2008	ARF	UK	2556	--	0-50	> 350	10.29	5.11, 20.98
Krawczyk, 2004	Cohort	1992-2002	CRF	USA	6361	--	< 200	200-349	4.3	2.1, 8.7
Roe, 2008	Cohort	1998-2005	ARF	UK	2274	8.0	< 100	> 200	6.75	2.5, 18.3
Roe, 2008	Cohort	1998-2005	ARF	UK	2274	8.0	100-199	> 200	3.02	0.99, 9.13
Szczech, 2002	Cohort	1994-1999	RF	USA	2057	4.5	< = 200	> 200	3.57	1.72, 7.14
Wei,	Cohort	1993-1997	HIVAN	USA	44	5.1	< = 100	> = 100	2.73	0.82, 9.14

**Table 2:** Details of age categories

Study name	Study type	Period	Disease	Location	study size	Follow up	Age (group 1) years	Age(group 2)years	RR	95%
Campbell, 2009 [10]	cross-sectional	1998-2005	CKD	BT, UK	3439	--	> = 50 (Indinavir)	< 50	4.92	1.31, 18.4
Campbell, 2009 [10]	cross-sectional	1998-2005	CKD	UK	3439	--	> = 50 (Tenofovir)	< 50	5.42	1.71, 16.8
Crane, 2007 [4]	Cohort	2001-2006	Kidney dysfunction	WA, USA	445	--	30-40	< 30	0.90	0.2, 3.2
Crane, 2007 [4]	Cohort	2001-2006	Kidney dysfunction	USA	445	--	40-50	< 30	2.10	0.6, 7.6
Crane, 2007 [4]	Cohort	2001-2006	Kidney dysfunction	USA	445	--	> 50	< 40	4.40	1.1, 17.2
Franey, 2009 [13]	Cohort	2004-2007	RI	South Africa	2189	--	> 40	< 30	4.65	3.54, 6.10
Ganesan, 2010 [14]	Cohort	1986-2008	CKD	USA	4044	6.5	> = 35	< 35	2.6	1.7, 4.0
Lucas, 2007 [20]	Cohort	1988-2004	CKD	MD, USA	4509	4.6	30-49.9	< 30	1.50	0.7, 3.3
Lucas, 2007 [20]	Cohort	1988-2004	CKD	USA	4509	4.6	> = 50	< 50	2.00	0.8, 4.7

### Relative risk of kidney disease for late-stage HIV versus non-AIDS

We estimated the relative risk of renal disease among people in late-stage HIV infection (AIDS) compared to other PLHIV. We identified three studies [20,21,23] estimating relative risk of kidney disease among people who have AIDS symptoms compared to other PLHIV. Lucas et al. reported the incidence rate ratio of end-stage kidney disease (ESKD), defined as receiving renal replacement therapy for 51 HIV-infected people without AIDS compared with 125 people with AIDS to be 2.7 (1.9-4.0) [20]. The IRR was adjusted by age, sex, AIDS status and HIV treatment era. Another study, by Mocroft et al. [21] estimated the relative hazard of chronic kidney disease having eGFR ≤ 60 ml/min per 1.73 m2 for ≥ 3 months among 6843 HIV-positive people to be 2.22 (1.14-4.32) after adjusting for age, sex, diabetes, hypertension and other risk factors. Roe et al. [23] estimated the relative risk of CKD stage 3 among 2274 HIV-infected people to be 6.72 (3.4-13.3), adjusting for major confounding factors. We note that this study classified the episodes of ARF into two onsets of action after initiating HIV care; we included the estimate of early onset of CKD stage 3 to avoid confounding with other co-infections in the late-onset of CKD. The pooled relative risk of CKD among people with late-stage HIV infection was 3.32 (1.86-5.93) compared to other PLHIV. There was statistically significant evidence of heterogeneity among these studies (I-squared 69.3%, p = 0.039) (Figure 2f).

### Relative risk of renal disease with age

We investigated whether the risk of renal disease depends on age. We identified five relevant studies that estimated the relative risk of renal disease for every 10 year increment in age where the reference group was age-matched HIV-negative subjects [2,7,11,19,21]. We found a clear association of increased risk of renal disease with increasing age (Figure 2g). The pooled relative risk of renal disease among PLHIV per 10 year increase in age was found to be 1.54 (1.16-2.05). The heterogeneity of this outcome was estimated as I-squared = 87.6% (p < 0.001).

Other studies also link age to the relative risk of renal disease among PLHIV but did not report age groups that could be incorporated into comparable estimates for our meta-analysis [4,10,13,14,20]. Campbell reported 81 CKD stage 3 patients among 3439 PLHIV with GFR < 60 mL/min for ≥ 3 months [10]. He estimated the relative risk among patients aged ≥ 50 years initiating IDV/TDF to be 4.92 (1.31-18.4) and 5.42 (1.71-16.8), respectively, when compared with patients aged less than 50 years [10]. Crane estimated the relative risk of 51 CKD stage 3 patients among 445 HIV-infected people who were aged 30-40 years, 40-50 years and over 50 years compared to people less than 30 years to be 0.90 (0.2-3.2), 2.10 (0.6-7.6) and 4.40 (1.4-6.8) respectively [4]. The other cohort studies [13,14,20] provided similar trends across age and CD4 counts. We found that the risk of renal disease among HIV-infected people was positively correlated with age (correlation, r = 0.71). The meta-analysis determined that relative risk of renal disease in HIV-infected people by 10-year increment in age was 1.54 (Figure 2h).

### Meta-regression analysis

We performed univariate meta-regression to explore factors that might account for heterogeneity between the relative risk of renal disease and the study characteristics. Potential explanatory covariates considered were study design, study period, duration of follow-up, diseases, study location, study size, race/ethnicity and estimated glomerular filtration rate (eGFR) type. We found that geographic location, type of disease and eGFR methods were significantly associated with the relative risk of renal disease.

### Sub-group outcomes

We performed three subgroup analyses based on the results of meta-regression. We found that the adjusted relative risk of renal disease associated with TDF was 2.01 (1.3-3.1) with a moderate amount of heterogeneity 53.7% (p = 0.09)(which decreased from previously calculated heterogeneity of 78.4% (p = 0.001)) caused by geographic location and eGFR. The type of disease reported causes heterogeneity between studies in the analysis of the effect of ART. Our resultant subgroup analysis by type of disease (HIVAN) changed the estimates from a relative risk of 0.54 (0.29-0.99) with heterogeneity of 80.5% (p < 0.001) to 0.42 (0.18-0.97) with heterogeneity of 60.4% (p = 0.081). We also found that type of disease (CKD) reported contributed to heterogeneity of the estimates for every 10-year increment in age. Our subgroup analysis by type of disease changed the estimates from a relative risk of 1.54 (1.16-2.05) with heterogeneity of 87.6% (p < 0.001) to 1.49 (1.29-1.71) with null heterogeneity of 0.0% (p = 0.390). It is noted that biopsy-proven HIV-associated nephropathy (HIVAN) was not included in the classification of disease in this subgroup analyses.

### Publication bias

We found no evidence of publication bias in our analyses. For example, among studies comparing relative risk of renal disease between HIV-infected and uninfected people, there was no evidence of publication bias by funnel plot symmetry, Egger's p = 0.757. We found similar estimates in other estimates with no significant evidence of publication bias. However, other studies may have been conducted to calculate the relative risk of renal disease that were not published and identified in our search methods.

## Discussion

We conducted a systematic review and series of meta-analyses to calculate the pooled relative risk of renal disease for PLHIV across available sources of evidence. Our analysis suggests that PLHIV have increased risk of renal disease. Specifically, the relative risk of renal disease for PLHIV was found to be 3.87 times greater than in HIV-uninfected people. The relative risk of renal disease for late-stage HIV infection (AIDS) was found to be 3.32 times more than that of PLHIV at earlier stages of infection. The relative risk of renal disease among PLHIV treated with ART was found to be decreased by 46% compared to treatment-naïve PLHIV. This indicates that ART could have a protective effect against renal disease in PLHIV. We also found that the risk of renal disease for HIV-infected people receiving TDF-based treatment was found to be 56% greater than the risk for HIV-infected people who are treated with non-TDF-based ART.

Our estimates are consistent with earlier reviews of specific associations with renal disease. A Multicentre AIDS Cohort Study by Palella et al. [35] found the relative risk of proteinuria with GFR decreased rate was 5.0 (p < 0.001) among PLHIV without AIDS compared to HIV-uninfected people and 2.18 (p = 0.02) among people with AIDS compared to other PLHIV, respectively. A review of TDF conducted by Cooper et al. found a non-significant effect of TDF-based ART of 0.7% (0.02-1.2) [36]. We also found that the duration of exposure to ART and age are important contributors to the risk of acquiring renal disease: specifically, each year of ART increased the relative risk by an estimated 29% and each 10-year increment in age increased the risk of renal disease by 54%.

One study identified in our search strategy, by Crane et al. [4], did not have similar comparator groups with other studies and thus could not be pooled in these estimates. The study reported estimates in various age groups and within ART groups. They found the risk of CKD among people receiving tenofovir was greater if the patients also received didanosine and amprenavir antiretrovirals, were of greater age, and lower baseline weight [4].

The underlying reason for development of CKD in HIV-infected patients is likely that HIV can cause direct injury to the kidneys as manifested by HIVAN [37]. The CKD can be developed by drug-induced nephrotoxicity to prevent HIV-infection, dependent on exposure to antiretroviral drug regimen. TDF-induced regimens are more likely to increase CKD than non-TDF-based regimen. There are other mechanisms of developing CKD among PLHIV as described in a recent study [37].

In our analyses we attempted to eliminate bias and confounding wherever possible. Individual studies controlled for certain confounders between the treatment and control groups but not all studies controlled for the same variables. Due to differences between study categorizations it is possible that our analysis may have some bias due to misclassification error. This may be particularly relevant for comparisons between HIV-infected people receiving ART versus treatment-naïve people because some of the people with unknown treatment exposure could have been classified as treatment-naïve. It is possible that there are other important characteristics beyond the effects of antiretroviral drugs that differ between populations of people who are given and take ART and those who are not treated. We were unable to conduct an analysis based on duration of ART, which may be an important determining factor. For individual studies in which there was some uncertainty in definitions of populations in any arm we conducted a sensitivity analysis by performing the meta-analysis without the questioned study, but we found our pooled estimates to be relatively robust. Our meta-analysis ended up with relatively small numbers in each grouping with reasonable amounts of heterogeneity. However, estimates from our meta-analyses provided no significant evidence of publication bias. Our effect measures were relatively consistent among the trials. Also, abstracts did not provide final, peer-reviewed, data and that the GFR equations have not been validated in HIV populations. Given these potential limitations, we believe our pooled estimates are accurate indications of the relative risk of CKD for HIV-infected people based on the available empirical evidence. However, it is important to note the importance of race/ethnicity, as it pertains to HIV-associated kidney disease. We were unable to conduct sub-analyses by race. However, a number of studies reported that people of African descent among HIV-infected population known to have high risk, as HIV-associated nephropathy (HIVAN) occurs disproportionately among those of African descent [9]. hepatitis C co-infection is also associated with kidney disease which was not included in our analysis [38].

## Conclusion

Although the health and survival of PLHIV has improved with effective antiretroviral therapies, HIV-infected people are at substantially greater risk of developing other co-morbidities, such as CKD, compared to uninfected people. This is a significant issue for populations of PLHIV, particularly as they get older and become more treatment experienced. Increasingly, HIV-positive populations will require long-term clinical management of numerous conditions along with their HIV infection. CKD is likely to be an important condition to be confronted in the future in populations of PLHIV.

## Competing interests

The authors declare that they have no competing interests.

## Authors' contributions

FMI conducted the review of literature, extracted data, performed all analyses and wrote the first draft of the manuscript; JW reviewed literature and assisted with analyses; JJ provided strategic advice and assisted with editing the Manuscript; DPW supervised the project and interpreted results and assisted with writing the manuscript. All authors read and approved the final manuscript.

## Pre-publication history

The pre-publication history for this paper can be accessed here:

http://www.biomedcentral.com/1471-2458/12/234/prepub
